# The role of ubiquitination and deubiquitination in the regulation of cell junctions

**DOI:** 10.1007/s13238-017-0486-3

**Published:** 2017-10-27

**Authors:** Junting Cai, Miranda K. Culley, Yutong Zhao, Jing Zhao

**Affiliations:** 1 Acute Lung Injury Center of Excellence, Division of Pulmonary, Asthma, and Critical Care Medicine, Department of Medicine, The University of Pittsburgh, Pittsburgh, PA 15213 USA; 2 Xiangya Hospital of Central South University, Changsha, 410008 China

**Keywords:** cell-cell junctions, protein stability, ubiquitination, deubiquitination, Rho GTPases

## Abstract

Maintenance of cell junctions plays a crucial role in the regulation of cellular functions including cell proliferation, permeability, and cell death. Disruption of cell junctions is implicated in a variety of human disorders, such as inflammatory diseases and cancers. Understanding molecular regulation of cell junctions is important for development of therapeutic strategies for intervention of human diseases. Ubiquitination is an important type of post-translational modification that primarily regulates endogenous protein stability, receptor internalization, enzyme activity, and protein-protein interactions. Ubiquitination is tightly regulated by ubiquitin E3 ligases and can be reversed by deubiquitinating enzymes. Recent studies have been focusing on investigating the effect of protein stability in the regulation of cell-cell junctions. Ubiquitination and degradation of cadherins, claudins, and their interacting proteins are implicated in epithelial and endothelial barrier disruption. Recent studies have revealed that ubiquitination is involved in regulation of Rho GTPases’ biological activities. Taken together these studies, ubiquitination plays a critical role in modulating cell junctions and motility. In this review, we will discuss the effects of ubiquitination and deubiquitination on protein stability and expression of key proteins in the cell-cell junctions, including junction proteins, their interacting proteins, and small Rho GTPases. We provide an overview of protein stability in modulation of epithelial and endothelial barrier integrity and introduce potential future search directions to better understand the effects of ubiquitination on human disorders caused by dysfunction of cell junctions.

## Introduction

Cellular junctions are connections between neighboring cells or between a cell and the extracellular matrix. Adherens junctions (AJs) and tight junctions (TJs) are principal components of cell-cell junctions that control the paracellular transport of other cells and proteins, and ultimately maintain normal barrier function. AJs contain a group of singular transmembrane proteins called cadherins which indirectly connect with cytoskeleton (Cadwell et al., 2016). TJs are comprised of a number of transmembrane proteins, including occludin, claudins, junction adhesion molecules (JAMs), and endothelial cell selective adhesion molecules (ESAMs) (Van Itallie and Anderson, 2014). Zonula occludens (ZO) proteins, ZO-1, -2 and -3, link these transmembrane proteins to actin. TJs also serve as a barrier and allow for selective molecular transportation (Hartsock and Nelson, 2008).

Expression levels of intracellular proteins are controlled by processes of synthesis and degradation. Ubiquitin (Ub), a small regulatory protein, is conjugated to specific proteins, marking these targets for degradation in the Ub-proteasome system (UPS) or the lysosomal system. The process of ubiquitination requires three enzyme groups: Ub-activating enzyme E1, Ub-conjugating enzyme E2, and Ub ligase E3. Thus far, ~600 Ub E3 enzymes have been identified and are responsible for ligating Ub or Ub chains to certain substrates (Komander and Rape, 2012). Deubiquitinating enzymes (DUBs) serve to remove Ub from ubiquitinated proteins, thus stabilizing the substrates. The reversible regulation by Ub E3 and DUBs occurs at cell junctions, and has received increasing attention as a way to understand the modulation of barrier function in the context of diseases (Schaefer et al., 2012).

In this review, we will introduce the components of the intercellular junctions and highlight the roles of ubiquitination and deubiquitination systems in regulation of AJs, TJs, and their related proteins including Rho GTPases. We will discuss future directions focusing on the strategies to better understand the proteolysis in epithelial and endothelial barrier integrity.

## Components in Intercellular Junctions

Barrier integrity is primarily maintained by intercellular junctions, which in turn control the paracellular transport of proteins, fluids, and small molecules. There are three major types of cell junctions: AJs, TJs, and gap junctions. Each junction consists of multiprotein complexes that allow for cell-cell or cell-extracellular matrix contact. Here, we will majorly focus on AJs and TJs.

### Adherens junction proteins

AJs are localized below TJs and perform multiple functions including initiating TJ assembly (Capaldo and Macara, 2007), stabilizing cell-cell adhesion, modifying cytoskeleton rearrangement, signaling transduction, and transcriptional regulation. In endothelial cells specifically, AJs direct endothelial barrier permeability and their dysfunction leads to interstitial edema and hemorrhage (Dejana et al., 2008). Similar to endothelial cells, epithelial cells’ AJs contribute to maintaining epithelial barrier integrity (Mezzano et al., 2014). Loss of AJ integrity has been linked to tumor stage (Zhou et al., 2015).

**Figure 1:**
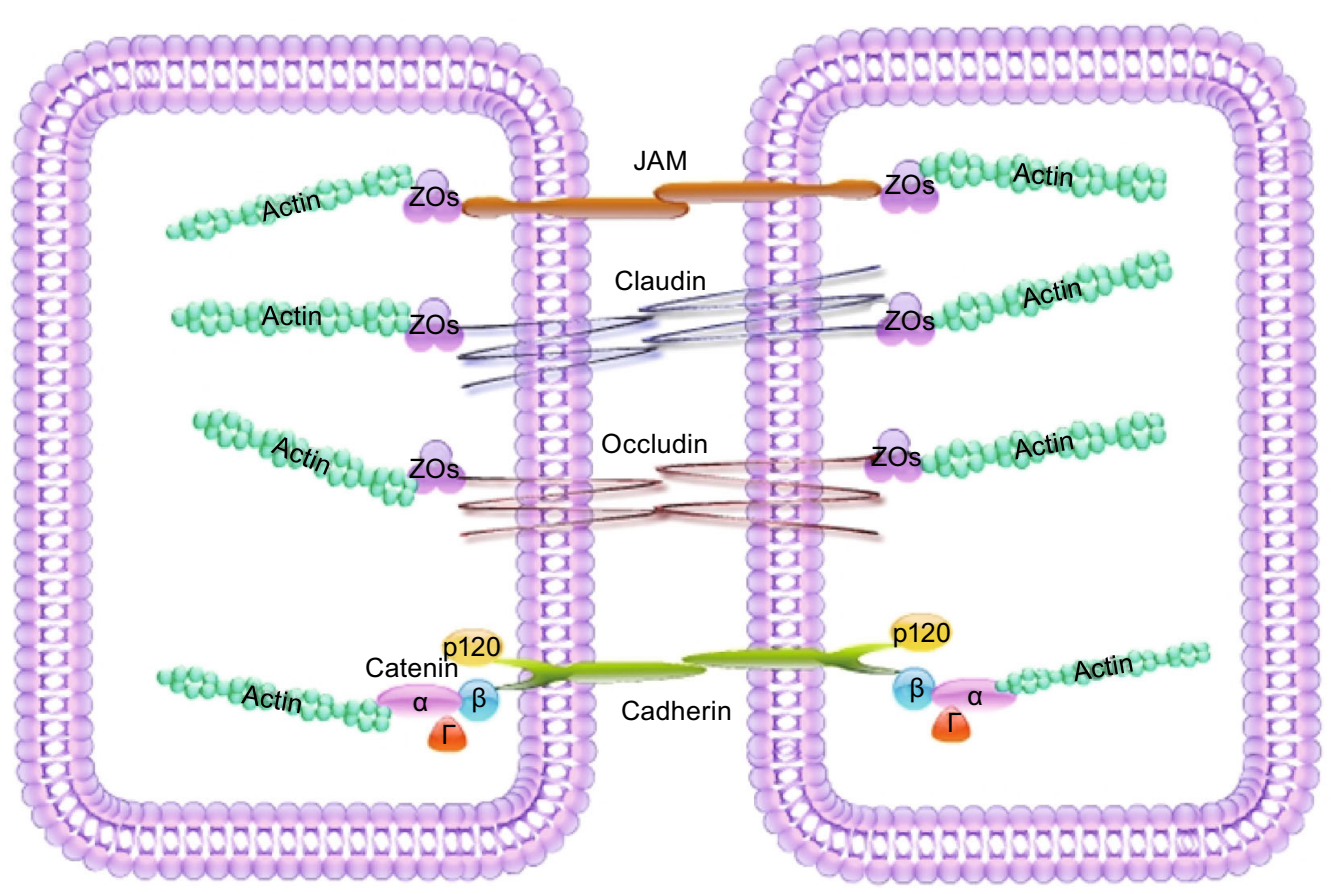
**Molecular components of intercellular junctions**. Adherens junctions (AJs) are composed of cadherins and catenins (α-, β-, γ- and p120-catenin). α-Catenin indirectly binds to cadherins via β-catenin or γ-catenin and physically links the complex AJs to the actin cytoskeleton. Tight junctions (TJs) are composed of claudins, occludin, and JAMs. ZOs link with tight junction proteins to actin

Cadherin, a Ca^2+^-dependent glycoprotein with a single transmembrane domain, is indispensable for AJ function. The cadherin family consists of several tissue-specific forms including epithelial (van Roy and Berx, 2008), vascular endothelial (Lampugnani et al., 1992), neurons, and placenta cadherins (E-, VE-, N- and P-cadherin, respectively) (Vieira and Paredes, 2015). Cadherins form intercellular junctions by pairing with extracellular domains of cadherins on the neighboring cells (Brasch et al., 2012). Cadherin also has a conserved cytoplasmic tail with a juxtamembrane domain (JMD) that binds to p120-catenin, and a catenin binding domain (CMD) that binds to β-catenin (Fig. 1).

Catenins, a family of proteins composed of α-, β-, γ- and p120-catenin, are another critical part of AJs that link cadherins to actin and contribute to barrier integrity. Both β- and p120-catenin bind to cadherin via their armadillo repeats (Hartsock and Nelson, 2008). β-Catenin binds with α-catenin and IQGAP in addition to cadherin (Hartsock and Nelson, 2008; Tian et al., 2011). The E-cadherin/β-catenin interaction is required for cadherin to exit the endoplasmic reticulum (Chen et al., 1999). Unlike β-catenin, p120-catenin only binds with the JMD to help stabilize cadherin at the plasma membrane (Conacci-Sorrell et al., 2002) and to regulate cell mobility (Kourtidis et al., 2013). Further, α-catenin has been shown to link the cadherin/β-catenin complex to F-actin (Rimm et al., 1995).

### Tight junction proteins

TJs are formed at the apical side of the intercellular cleft and control the movement of ions and solutes between adjacent cells via the paracellular pathway. TJs are composed of claudins, JAMs, and tight junction-associated MARVEL proteins (TAMPs). The TAMP family includes occludin, tricellulin, and MarvelD3. The zonula occludens (ZOs), also called tight junction proteins (TJPs), are members of the membrane-associated guanylate kinase (MAGUK) homologue family of proteins. They link the intracellular domains of the transmembrane strands, especially occludin and claudins, to actin. Both occludin and claudins are integral plasma membrane proteins that have four transmembrane domains and form two extracellular loops (Furuse et al., 1993; Krause et al., 2008).

Occludin was the first identified transmembrane protein in TJs and contains two extracellular loops flanked by cytoplasmic C-termini. The extracellular loops are responsible for localization of occludin in the TJs. The cytosolic C-terminal domain interacts with cytoplasmic proteins such as ZOs while the N-terminal domain mediates TJ sealing. The cytosolic C-terminal domain is enriched in serine, threonine, and tyrosine residues, which are subjected to various modifications. Changes in occludin expression and distribution are associated with histological abnormalities in various diseases, including particular cancers (Tobioka et al., 2002, 2004), diarrhea (McNamara et al., 2001), ulcerative colitis (Kucharzik et al., 2001), and HIV-associated encephalitis (Dallasta et al., 1999).

Claudins are recognized as the most important component in TJs. In humans, there are 26 claudins with molecular weights ranging from 21 to 34 kDa. Claudins are expressed in a tissue-specific manner resulting in specific barrier characteristics. For example, claudin-5 is predominantly expressed in endothelial cell tight junctions and plays an important role in the formation of the blood-brain barrier (Morita et al., 1999). Expression of claudins 1, 3, 4, 5, 7, 8, 10, 15, and 18 have been identified in lung epithelial tissues (Wang et al., 2003a; Coyne et al., 2003; Kaarteenaho-Wiik and Soini, 2009; Niimi et al., 2001; Rokkam et al., 2011). Abnormal expression or distribution of claudins contributes to many pathological conditions such as inflammatory bowel disease, inflammatory diseases of lung and central nervous system (CNS), diabetic retinopathy, nephrocalcinosis, progressive renal failure, tumorigenesis, and metastasis (Zeissig et al., 2007; Felinski and Antonetti, 2005; Konrad et al., 2006; Coyne et al., 2002; Rubin and Staddon, 1999; Soler et al., 1999).

Tight junction proteins, ZOs, link occludin and claudins to the actin cytoskeleton. All three ZO proteins directly interact with the extended C-terminus of occludin and claudins. ZOs contain a conserved core of protein-binding motifs on its N-terminus, including a Src homology 3 (SH3) module, a guanylate kinase-like (GuK) domain, and three PSD-95/discs-large/Zonula occludens (PDZ) domains. The SH3-GuK region and the adjacent unique domains 5 and 6 in the N-terminal domains of ZO proteins link all transmembrane proteins in the TJs. Besides assembling TJs, ZO proteins regulate AJs by binding cadherin and catenin at an early stage of AJ formation (Kausalya et al., 2004; Fanning and Anderson, 2009). The diverse binding potential of ZOs is just one example of how cell junction components are dynamically dependent upon one another for functional integrity.

### Regulation of junctional proteins recycling and remodeling

**Figure 2:**
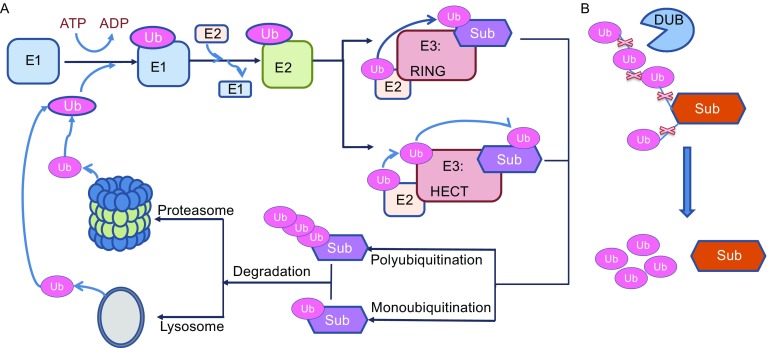
**Protein ubiquitination and deubiquitination**. (A) E1 starts the ubiquitination process along with ATP. The E1 enzyme passes the Ub protein to E2s. The E2s are then complex with Ub ligase E3s. In the final step, E3s catalyze Ub transferring to substrates. Proteins can be ubiquitinated with multiple types including monoubiquitination and polyubiquitination, and degraded in the lysosome or the proteasome, respectively. (B) DUBs remove Ub molecules from target substrates

There is constant trafficking and recycling of intercellular junction proteins between the cytoplasm and the cell surface. The recycling system maintains the balance of intercellular junction proteins on the plasma membrane and recognizes inappropriately internalized junction proteins for degradation. Ultimately this provides a dynamic way to rapidly regulate paracellular permeability in response to physiological variations. Furthermore, disruption of recycling alters junction proteins at the plasma membrane and this disruption has been associated with tumor formation and inflammatory diseases (Brennan et al., 2010; Utech et al., 2010). Endocytosis is specific part of intercellular junctions remodeling and recycling. Post-translational modifications such as ubiquitination, phosphorylation, and palmitoylation regulate endocytosis and post-endocytic sorting (Welling and Weisz, 2010; Cummins, 2012; Traweger et al., 2002). Recent studies demonstrate a critical role of ubiquitination in the regulation of cell junction proteins internalization and degradation. However, there were no review articles to summarize these studies. In this review, we overview recent findings regarding the Ub-dependent trafficking and turnover of junction proteins and the implications in pathological conditions such as cancer and inflammatory diseases (Fig. 2).

## Ubiquitination and Protein Degradation

**Figure 3:**
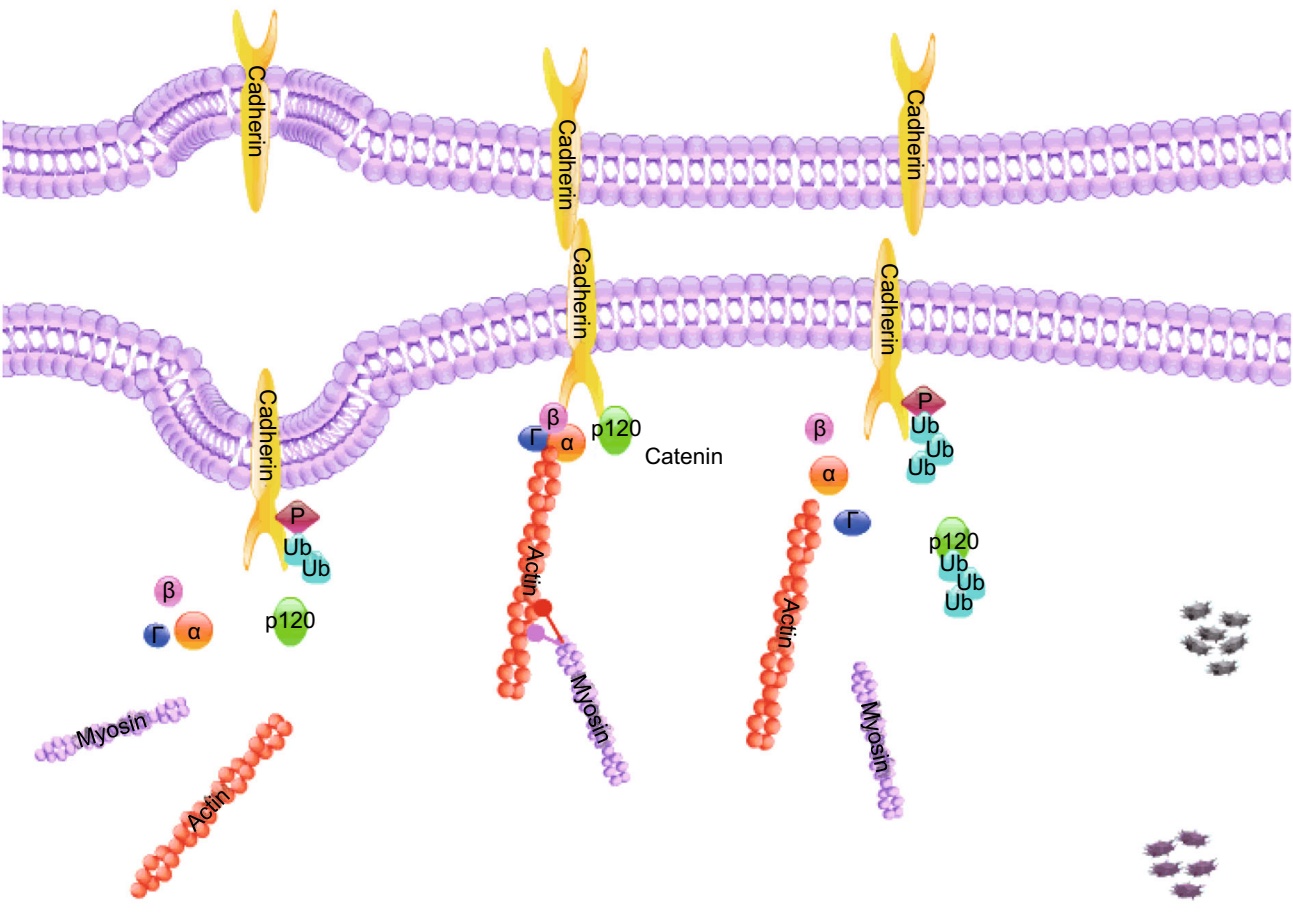
**Schematic diagram of the cadherins endocytosis, degradation, and recycling**. Phosphorylation triggers polyubiquitination of cadherin, which can be reversed by deubiquitinating enzymes. Ubiquitinated cadherin is degraded in the lysosome. Its linker, p120 catenin is ubiquitinated and degraded in the proteasome

Ub is a 76-amino acid protein that is ubiquitously expressed in all tissues. Ubiquitination is the process of ligating Ub molecule(s) to a substrate protein, in turn regulating protein stability and translocation. Ubiquitination is an ATP-dependent three-step enzymatic cascade reaction requiring E1, E2, and E3 ligases. Ub E3 ligase conjugates Ub to lysine (K) residues of the substrate. Proteins can be ubiquitinated at one or multiple K residues with either a single ubiquitin molecule (mono- or multi-monoubiquitination) or a chain of ubiquitin (polyubiquitination). A Ub chain is formed by linking Ub molecules through one of their lysine residues (K6, K11, K27, K29, K33, K48, and K63) or the N-terminal methionine residue (M1), which results in Ub chain structure diversity and specificity (Behrends and Harper, 2011). Monoubiquitination is involved in transcriptional regulation, DNA damage repair, and membrane-associated endocytosis (Nakagawa and Nakayama, 2015). Among the existing types of polyubiquitin chain linkage, K48 linkages are the most abundant Ub chain type (Xu et al., 2009) and K48-linked polyubiquitin chains are a canonical signal for protein degradation in the proteasome (Chau et al., 1989; Thrower et al., 2000). In this review, we will focus primarily on the roles of ubiquitin-proteasome system (UPS) in regulating cell junction proteins (Fig. 3).

Deubiquitinating enzymes (DUBs) are a large set of proteases that remove Ub molecules from target substrates to reverse ubiquitination (Wilkinson, 2000). So far, more than 100 DUBs have been identified and classified into two main groups: cysteine proteases and metalloproteases. There are 4 types of cysteine proteases: Ub-specific proteases (USPs), Ub C-terminal hydrolases (UCHs), ovarian tumor proteases (OTU), and Machado-Josephin domain proteases (MJDs) (Heideker and Wertz, 2015). The metalloproteases are named for their requirement of zinc for catalysis and contain the Jab1/Mov34/Mpr1 Pad1 N-terminal+ (MPN+) (JAMM) domain proteases (Bonnet et al., 2008). The balance between the Ub E3 ligases and DUBs activity is crucial for not only regulating protein stability but also for maintaining the free Ub pool (Coyne and Wing, 2016). An imbalance has been linked to various types of cellular dysfunction in controlling of transcellular transportation, barrier integrity, intracellular signaling, and gene transcription (Singh and Singh, 2016; Qiu et al., 2017). Next, we will review the role of Ub E3 ligases and DUBs in the regulation of key proteins in cell junctions.

## Ubiquitination of Adherens Junction Proteins

### Regulation of cadherin stability and expression by Ub E3 ligases: direct and indirect effects

As a principal adhesion molecule, cadherins are under constant regulation in order to maintain a balance between stability and plasticity of the cellular barrier (Cadwell et al., 2016; Aparicio et al., 2012). Hakai, an Ub E3 ligase, targets tyrosine phosphorylated E-cadherin, regulating its endocytosis and stability through ubiquitination (Fujita et al., 2002). The Hakai-mediated E-cadherin ubiquitination site is located in its JMD, which also contains a p120-catenin binding site. Phosphorylation by an activated tyrosine kinase enhances E-cadherin binding with Hakai and prevents E-cadherin binding with p120-catenin, leads to E-cadherin ubiquitination and endocytosis (Fujita et al., 2002). In epithelial tumor progression as well as some process of normal tissue development, E-cadherin is transported to the lysosome for degradation instead of being recycled (Palacios et al., 2005). Rab5 and Rab7, members of Rab GTPase family, are believed to participate in the shuttling of ubiquitinated E-cadherin from endosomes to lysosomes in response to stimulation by growth factors, such as hepatocyte growth factor (HGF) and epidermal growth factor (EGF) (Palacios et al., 2005). Cadherin endocytosis is associated with disease development and progression. For example, Src-mediated tyrosine phosphorylation of E-cadherin and subsequent ubiquitination, endocytosis, and degradation occurs under pathological conditions such as decreased pH, bacterial invasion, and increased IFN-γ (Chen et al., 2009a; Bonazzi et al., 2008; Smyth et al., 2012), resulting in loss of epithelial integrity and increased epithelial permeability (Smyth et al., 2012; Bonazzi et al., 2008; Chen et al., 2009a). Src-dependent E-cadherin internalization is increased with shear stress in an oropharyngeal cancer cell line (Lawler et al., 2009) and a mouse model of UV irradiation-induced squamous cell carcinoma (Brouxhon et al., 2007). Paxillin, an important protein for cell-matrix adhesion, is also down-regulated by Hakai overexpression; however, Hakai-dependent paxillin down-regulation is proteasome-independent (Rodríguez-Rigueiro et al., 2011). However, not all cadherins have Hakai-binding tyrosine residues, including two primary endothelial cadherins, N-cadherin and VE-cadherin (Balsamo et al., 1996; Baumeister et al., 2005; Fujita et al., 2002). Therefore, Hakai-mediated down-regulation of cadherins may be restricted to the epithelial AJs. Notably, Hakai is not the only Ub E3 ligase that has been connected to AJs protein turnover. The Ub E3 ligases, Fbxl20 and MARCH8, also ubiquitinate and reduce levels of E-cadherin on the cell surface in zebrafish embryos and in human colorectal cancer (Zhu et al., 2014; Kim et al., 2014). It is common that one substrate can be modulated by multiple Ub E3 ligases, while the expression patterns of Hakai, Fbxl20, and MARCH8 in organs have not been reported. The different biological effects of these Ub E3 ligases are still not clear.

E-cadherin expression is also regulated by transcriptional repressors, such as Vimentin, Slug, Snail, DeltaEF1, Zeb1, and Twist (Batlle et al., 2000; Schmidt et al., 2005; Eger et al., 2005; Vannier et al., 2013; Vesuna et al., 2008), which can be regulated by UPS system. Ub E3 ligases, Pellino-1, UBE3C, and Fbxo32 have been shown to upregulate Vimentin, Slug, and Snail, therefore resulting in the suppression of E-cadherin mRNA levels (Jeon et al., 2017; Tang et al., 2016; Tanaka et al., 2016). In addition, Ub-like with PHD and RING finger domain 1 (UHRF1), a member of a subfamily of RING-finger type Ub E3 ligases, is highly expressed in many human cancers, which suppresses the expression of E-cadherin through targeting retinoblastoma 1 (Rb1), thus promoting epithelial-to-mesenchymal transition (EMT) in human osteosarcoma cells (Unoki et al., 2010; Liu et al., 2016). Another study from Jung Y-D et al. exhibits that UHRF1 increases E-cadherin transcript by inducing transcriptional factors, Zeb1 and Snail, degradation (Jung et al., 2015). The different effects of UHRF1 on E-cadherin expression may due to different cancer cell types. There are a number of Ub E3 ligases, including Aryl hydrocarbon receptor (AHR) and Fbxo11, that upregulate cadherins expression by reducing transcription repressors, such as Snail (Li et al., 2017; Jin et al., 2015). Ubiquilin 1 contains a N-terminal Ub-like domain (UBL) and a C-terminal Ub-associated domain (UBA) and guides the ubiquitinated proteins for degradation by the proteasome (Ko et al., 2004; Shah et al., 2015). Ubiquilin 1 degrades Zeb1 or Snai1, resulting in increases of E-cadherin expression (Jung et al., 2015; Shah et al., 2015). The effects of these Ub E3 ligases on E-cadherin protein levels are indirect, thus modulation of these enzymes may influence other genes related to the targeted transcriptional factors. Compared to the E3 ligases that directly catalyze E-cadherin ubiquitination, targeting these Ub E3 ligases, such as UHRF1, Fbxo11, and Ubiquilin 1 may lead to unexpected side effects.

MDM2 is an Ub E3 ligase that can both directly and indirectly regulate E-cadherin levels. It has been shown that MDM2 interacts with E-cadherin and regulates its ubiquitination and degradation in the UPS (Yang et al., 2006). In addition, MDM2 plays a role in the regulation of E-cadherin transcriptional repressors, Slug and Snail. Low MDM2, high Slug, and low E-cadherin expression correlate with poor prognosis and early metastasis in non-small-cell lung cancer (NSCLC) patients (Wang et al., 2009). MDM2 controls the tumor suppressor p53 as well as p53-dependent Slug and Snail ubiquitination and degradation by the proteasome system. MDM2 upregulates expression of Snail, inducing EMT in breast cancer cells *in vitro* and *in vivo* by downregulating E-cadherin (Lu et al., 2016; Yang et al., 2006). Thus, many efforts have been focused on targeting MDM2 for cancer therapy, however, the effects of the strategy on other diseases, alveolar epithelial barrier function in the context of lung injury, have not been studied.

VE-cadherin, a principal adhesion molecule in the endothelium, can be ubiquitinated by the MARCH family Ub E3 ligase K5 (Mansouri et al., 2008). K5 is expressed in endothelial-derived tumor Kaposi sarcoma that caused by human herpesvirus 8 infection (Goto et al., 2003). Similar to E-cadherin, K5-induced VE-cadherin endocytosis requires simultaneous inhibition of p120-catenin and cadherin interaction (Nanes et al., 2017). It will be interesting to know whether the E3 ligase K5 plays a role in endothelial barrier integrity.

### Regulation of catenins stability and expression by Ub E3 ligases

p120-catenin plays an essential role in maintenance of the integrity of both AJs and TJs. Interaction with p120-catenin is a key regulatory step for stabilization of multiple cadherins on the cell surface. Recent studies have demonstrated that reduction of p120-catenin correlates with the progression of different human tumors (Jin et al., 2015; van Hengel and van Roy, 2007). Further, p120-catenin protein expression was rapidly decreased in LPS-challenged mouse lungs and inversely correlated with the severity of inflammation (Wang et al., 2011a). p120-catenin can be regulated by either calpain 1-mediated degradation or phosphorylation-dependent ubiquitination and proteasomal degradation (Ohno et al., 2007; Kusaba et al., 2010; Wang et al., 2011b), however, whether the Ub E3 ligases are responsible for p120-catenin have not been revealed.

As mentioned above, the recruitment of β-catenin to the plasma membrane by cadherins is crucial for AJ formation and stabilization (Huber and Weis, 2001; Lickert et al., 2000). Similar to p120-catenin, β-catenin protects the cadherin cytoplasmic domain from degradation by blocking sites that overlap with critical binding sites (Huber et al., 2001). β-Catenin is ubiquitinated by the Ub E3 ligase β-transducin repeat-containing protein (β-TrCP) and then degraded by the proteasome. β-TrCP specifically recognizes the phosphorylated β-catenin; phosphorylation is mediated by GSK3β/Casein kinase 1 (CK1) (Sadot et al., 2000; Amit et al., 2002; Hart et al., 1999). Inhibition of CK1 stabilizes AJs, while CK1 over-expression disrupts AJs (Fannon et al., 1995). FAS-associated factor 1 (FAF1) acts as a scaffold protein that promotes β-TrCP-mediated β-catenin ubiquitination. Deletion of FAF1 results in increase of β-catenin (Zhang et al., 2012). An Ub-like modifier, FAT10, non-covalently binds β-catenin and prevent GSK3β-induced β-catenin degradation (Aichem and Groettrup, 2016). While β-TrCP is responsible for the regulation of cytoplasmic phosphorylated β-catenin ubiquitination and degradation (Kitagawa et al., 1999; Latres et al., 1999), the Ub E3 ligase Jade-1 is believed to ubiquitinate both the phosphorylated and non-phosphorylated β-catenin in the nuclei (Chitalia et al., 2008). Nuclear β-catenin is known to regulate gene expression. The effect of Jade-1 on cell junctions is not clear yet.

γ-Catenin (or plakoglobin) is highly homologous to β-catenin and directly binds to cadherin family members, linking them to the actin cytoskeleton (Cowin et al., 1986) in desmosomes and AJs. β-TrCP also interacts with γ-catenin and regulates its polyubiquitination. Unlike β-catenin, majority of γ-catenin was found in Triton X-100 insoluble fraction that forms the membrane-cytoskeleton system. This localization maintains γ-catenin stability by preventing its interaction with Ub machinery (Sadot et al., 2000).

One E3 ligase may regulate certain biological functions through targeting related multi-substrates. It is possible that the Ub E3 ligases for cadherins may also modulate catenins’ ubiquitination, since cadherins and catenins form protein complexes inside of the plasma membrane. It is not clear during epithelial or endothelial barrier disruption how these Ub E3 ligases are recruited to substrates and which substrate is degraded first. This information will be helpful for focusing on which Ub E3 ligase acts first.

## Ubiquitination and Tight Junction Proteins

TJs, in association with AJs, play a pivotal role in tissue integrity and barrier function such as the blood-brain and blood-retinal barriers (Harhaj and Antonetti, 2004). Occludin was the first identified integral membrane TJ protein and can be regulated by various types of modification (Furuse et al., 1993; Antonetti et al., 1999; Coëffier et al., 2010). Itch, a HECT domain-containing Ub E3 ligase, interacts with the N-terminus of occludin, resulting in occludin ubiquitination and degradation by the proteasome (Traweger et al., 2002; Murakami et al., 2009). The phosphorylation of occludin at Ser490 is required for ubiquitination by itch, which facilities VEGF-induced vascular endothelial cell permeability (Antonetti et al., 1999; Murakami et al., 2009). Increased proteasome-mediated degradation of occludin has been shown in patients with irritable bowel syndrome (IBS) (Coëffier et al., 2010). In addition, itch also participates in the regulation of Sertoli TJ dynamics throughout spermatogenesis (Lui and Lee, 2005). When Sertoli cell TJs are assembled, itch is decreased while the level of occludin is increased (Lui and Lee, 2005). In animal models of permanent brain ischemia and ischemia-reperfusion injury, the loss of occludin in the blood-brain or blood-retinal barriers is also associated with itch activation and itch-mediated occludin ubiquitination (Zhang et al., 2013; Muthusamy et al., 2014). Nedd4-2, an important regulator of epithelial sodium channel transport in the collecting duct of kidney, also ubiquitinates occludin, resulting in a delay in TJ formation (Raikwar et al., 2010). A recent study shows that the Ub E3 ligase MARCH3 regulates endothelial permeability in response to inflammatory factors by controlling the expression of occludin. MARCH3-silencing results in both the upregulation of occludin gene and the strengthening of cell-cell contacts (Leclair et al., 2016). Ub-conjugating enzyme E2 J1 (Ube2j1) is the only E2 that has been reported to associate with internalized occludin (Su et al., 2010). More details of the mechanisms for Nedd4-2, MARCH3, VEGF and Ube2j1-induced ubiquitination are yet-to-be determined. Moreover, how these enzymes cooperate in the context of regulation of epithelial and endothelial barrier function needs further investigation. The regulation of these Ub E3 ligases expression in inflammatory diseases and human tumors will be focused in the future study.

Claudins are major structural components of the TJ that determine the paracellular permeability of epithelial and endothelial cells (Farquhar and Palade, 1963; Bazzoni, 2006). Recent studies aimed to understand how ubiquitination regulates claudins endocytosis, localization, and degradation. Within the claudin family, only the ubiquitination of claudin-1, -2, -4, -5, -8, -16 have been characterized. Ligand of Numb-protein X1 (LNX1p80), the Ub E3 ligase, is implicated in ubiquitination and lysosomal degradation of claudin-1, -2, and -4 (Takahashi et al., 2009). Carbachol, a cholinergic agonist of muscarinic and nicotinic acetylcholine receptors (mAChRs and nAChRs) (Jones et al., 2012) significantly decreased the amount of claudin-4 protein without affecting other TJ components. Carbachol-induced phosphorylation of claudin-4 at S195 is required for claudin-4 ubiquitination and internalization. Claudin-5 is the primary claudin expressed in endothelial TJs and plays a major part in maintaining the blood-brain barrier (Morita et al., 1999; Nitta et al., 2003). Claudin-5 is degraded both in a Ub-proteasome-dependent manner and in the Ub-independent lysosomal pathways (Mandel et al., 2012). Claudin-5 is polyubiquitinated on lysine residue 199 and degraded by the proteasome. Kelch-like 3 (KLHL3), a member of cullin-really interesting new gene E3 Ub ligase (CRL) complex, directly interacts with claudin-8 and regulates its ubiquitination and degradation (Gong et al., 2015). The KLHL3 R528H mutant significantly reduces claudin-8 ubiquitination and results in higher expression level of claudin-8 (Gong et al., 2015; Hou, 2016). Further, knockdown of KLHL3 in the collecting duct cells of mice profoundly increases the TJ conductance of chloride. KLHL3 regulation of claudin-8 ubiquitination may contribute to the changes of electrolyte and blood pressure imbalances (Gong et al., 2015). The ubiquitination not only regulates proteolysis, but also is involved in claudins membrane trafficking (Asaka et al., 2011; Hicke and Dunn, 2003). Recent study demonstrates that the Ub E3 ligase PDZRN3 catalyzes claudin-16 mono-ubiquitination and translocalization, thus regulating paracellular Mg^2+^ permeability in kidney (Marunaka et al., 2017). Proteasome inhibitor treatment induces claudin-1 colocalization with Rab5a and EEA-1, markers of early endosomes in the cytoplasm. Rab5a is associated with proteasome-mediated claudin-1 localization at the plasma membrane (Asaka et al., 2011). E3 SUMO-protein ligase PIAS has been reported to interact with and reduce the levels of claudin-2. However, this process is not through ubiquitination, but rather a similar process named SUMOylation (Van Itallie et al., 2012). To better understand claudins turnover, identification of the Ub E3 ligases that target each claudin are important.

Even ZO proteins are critical regulators in TJs and AJs, the interaction between the Ub system and ZO proteins is not fully understood. In Japanese encephalitis viral (JEV)-infected astrocytes, barrier disruption is caused by ZO-1 and claudin-5 degradation (Chang et al., 2015). Further studies have demonstrated that JEV infection-induced expression of interleukin-6 (IL-6) activates the UPS by upregulating the Ub ligase E3 component n-recognin-1(Ubr-1) and subsequent ZO-1 ubiquitination and degradation (Chen et al., 2014). However, the regulation of ZO-2 and ZO-3 degradation by the UPS have not yet been studied.

Junction adhesion molecules (JAMs), another TJ protein, contain a transmembrane domain, an extracellular portion, and a short cytoplasmic tail. The JAMs are classified into JAM-A, -B and -C by structure, and they form TJs by homophilic or heterophilic bindings with each other (Bazzoni, 2003). So far, there has been less work on characterizing a link between JAMs and the UPS. One study used siRNA knockdown and co-immunoprecipitation technologies to demonstrate that TGF-β3 induced JAM-B degradation by the UPS (Zhang and Lui, 2015). Because the TGF-β-SMAD pathway has been characterized in several inflammatory diseases and cancers, further study about the interaction between JAM and Ub could be equally important.

## DUBs and Cell Junction Proteins

Among all the proteins in AJs and TJs, β-catenin is the best-characterized in the context of interaction with DUBs so far. Most of these studies have focused on the regulation of β-catenin levels in tumorigenesis. Ub-specific peptidase 4 (USP4) is found to hydrolyze both K48- and K63-polyubiquitin chains of β-catenin (Zhao et al., 2009). In a colon cancer cell line, USP4 deubiquitinates β-catenin and mediates its nuclear transportation, thus promoting Wnt/β-catenin signaling and cancer cells growth, migration, and invasion (Yun et al., 2015). Additionally, DUBs USP14, USP15, USP47, and Fam/USP9X have been reported to prevent β-catenin turnover by inhibiting its Ub-proteasomal degradation (Taya et al., 1999; Greenblatt et al., 2016; Zhang et al., 2017; Shi et al., 2015), but the direct interaction between USP14 and β-catenin is yet-to-be shown. UCHL1 is a member of the Ub C-terminal hydrolase family, which has been shown to regulate β-catenin expression in colorectal cancer (Zhong et al., 2012; Bheda et al., 2009). UCHL1 affects β-catenin levels by functioning as a transcriptional co-activator (Bheda et al., 2009). Collectively, while effects of these DUBs on cell junctions have not been reported, they are potential targets for anti-cancer therapeutics.

USP47 can be recruited to AJs and suppress the ubiquitination of E-cadherin, thus inhibiting its resultant proteasomal degradation (Sako-Kubota et al., 2014). It has been proposed that USP7 may reverse self-ubiquitination of the Ub E3 ligase MARCH7, thus stabilizing MARCH7 which has been known to regulate the expression levels of E-cadherin and β-catenin (Zhang et al., 2016). Desmosomes are spot-like adhesion junctions on the lateral side of plasma membranes. YOD1, a DUB from OTU family, deubiquitinates desmin. MicroRNA-21, a microRNA that plays a pivotal role in the cardiovascular diseases (Darabi et al., 2016), can inhibit YOD1’s function and disrupt desmosomes (Ye et al., 2014). Recent study demonstrates that USP48 regulates E-cadherin mRNA levels through stabilizing the TRAF2-JNK pathway in lung epithelial cells (Li et al., 2017). This study exhibits an indirect effect of DUBs on regulation of E-cadherin levels and lung epithelial barrier integrity.

Certain DUB family members have an inactive protease domain including USP53 (Komander et al., 2009). One study demonstrates that USP53 maintains the integrity of the auditory system by modulating the barrier properties. Instead of binding with Ub, USP53 interacts with ZO-1 and ZO-2 as a part of the tight junction complex (Kazmierczak et al., 2015).

Thus far, there is little research about DUBs regulation of TJs. The future challenges will be discoveries of DUBs that reverse ubiquitination of TJ proteins and enhance epithelial and endothelial barrier integrity. Like Ub E3 ligases, one DUB can target multi-substrates. It is possible the DUBs for AJs also modulate major components in TJs. Identification of TJ-related DUBs will be critical for understanding role of DUBs in maintenance of cell junctions.

## Rho GTPases as Cytoskeleton Regulators

**Figure 4:**
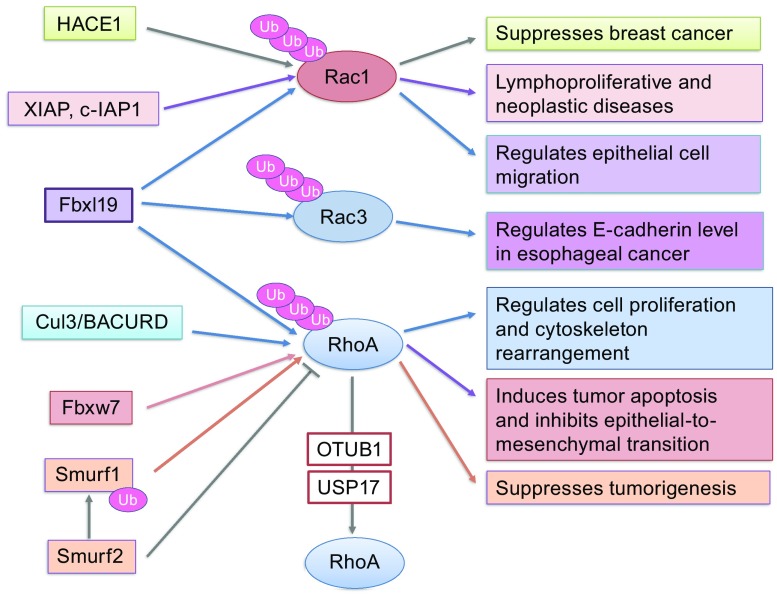
**Overview of ubiquitination and deubiquitination of Rho GTPases and their effects on Rho GTPases biological functions**. Rac1 is ubiquitinated by E3 ligases, HACE1, XIAP, c-IAP1, and Fbxl19. Rac3 is ubiquitinated by Fbxl19. RhoA is ubiquitinated by Fbxl19, Cul3/BACURD, Fbxw7, and Smurf1. Smurf2 negatively regulates Smurf1, thus stabilizing RhoA. OTUB1 and USP17 deubiquitinate and stabilize RhoA. Regulation of Rho GTPase stability controls tumorigenesis, cytoskeleton rearrangement, and cell migration

The Rho family is a unique subfamily of the Ras superfamily of small GTPases and is made up of 20 members, which controls actin cytoskeleton dynamics and cell movement (Bustelo et al., 2007; Boureux et al., 2007). Rac, Rho, and CDC42 are most well-studied members that are also involved in epithelial and endothelial barrier function (van Nieuw Amerongen et al., 2000; Timmerman et al., 2015; Kouklis et al., 2004). RhoA, in particular, has been shown to disrupt cell junctions by inducing stress fiber formation (Hirase et al., 2001; van Nieuw Amerongen et al., 2000; Mikelis et al., 2015). Therefore, interventions that down-regulate RhoA should benefit barrier enhancement. Several Ub E3 ligases have been identified to regulate RhoA ubiquitination including Smurf1, Cullin3/BACURD, Fbxl19, and Fbxw7 (Wang et al., 2003b; Chen et al., 2009b; Wei et al., 2013; Li et al., 2016). Smurf1, a HECT domain Ub ligase, targets active GTP-RhoA for ubiquitination at the specific cellular protrusion, which inhibits contractility and stress fiber formation, ultimately promoting tumor cell migration and invasion (Wang et al., 2003b; Kwon et al., 2013). In addition, the tumor suppressor RASSF1A prevents tumorigenesis via interacting with Smurf1 and promoting Smurf1-mediated ubiquitination of RhoA (Lee et al., 2016). Cullin3/BACURD catalyzes GDP-RhoA ubiquitination and degradation by the proteasome, and knockdown of Cullin3/BACURD Ub E3 ligase results in actin stress fiber abnormalities (Chen et al., 2009b). Fbxw7, an Ub E3 ligase containing F-box and WD repeat domain, suppresses gastric cancer cell motility and invasion by inducing the ubiquitination and degradation of both total RhoA and GTP-RhoA. It reduces GTP-RhoA levels at leading edge (Li et al., 2016; Wang et al., 2003b). HECT domain and ankyrin repeat containing E3 Ub ligase 1 (HACE1) specifically catalyzes the ubiquitination of active Rac1 and exhibits tumor-suppressor function (Goka and Lippman, 2015; Mettouchi and Lemichez, 2012). Thus far, Fbxl19 is the only Ub E3 ligase found to target multiple GTPases including RhoA, Rac1, as well as Rac3 (Wei et al., 2013; Dong et al., 2014; Zhao et al., 2013), therefore reducing cell proliferation, cell migration, and EMT. Phosphorylation is necessary for Fbxl19 targeting Rho GTPases. For example, AKT-mediated phosphorylation of Rac1 promotes Fbxl19-mediated ubiquitination and degradation (Zhao et al., 2013) (Fig. 4).

Inhibitors of apoptosis proteins (IAPs) are Ub E3 ligases which function as the endogenous inhibitors of caspases. XIAP and c-IAP1 modulate Rac1 ubiquitination and degradation by the proteasome (Oberoi et al., 2012; Oberoi-Khanuja and Rajalingam, 2012). Downregulation of c-IAP1 and XIAP are associated with lymphoproliferative disorders and a severe and rare type of inflammatory bowel disease (Deveraux et al., 1997, 1998). Many studies demonstrate that IAPs are involved neoplasia, making them an attractive therapeutic target for cancer treatment (Schimmer, 2004). Whether phosphorylation contributes to XIAP- or c-IAP1-catalyzed ubiquitination of Rac1 is not clear. Future studies will reveal the molecular mechanisms of XIAP- and c-IAP1-regulated Rac1 ubiquitination and whether Fbxl19, XIAP, or c-IAP1 catalyzes ubiquitination of Rac1 on the same lysine residue.

There are two identified DUBs that regulate RhoA stability: OTUB1 and USP17. By forming a protein complex, OTUB1 stabilizes active RhoA and promotes tumorigenesis and prostate cancer cell invasion (Iglesias-Gato et al., 2015; Edelmann et al., 2010). USP17 is reported to regulate subcellular relocalization of GTPase RhoA, Rac, and CDC42 via an unclear mechanism (de la Vega et al., 2011).

Taken together with all these studies, we conclude that the ubiquitination and deubiquitination systems play a pivotal role not only in the direct interaction with AJ and TJ components, but also in the interaction with Rho GTPases that adjust the actin cytoskeleton. The latter is involved in the number of cancer and infectious disease processes, making it an interesting target for future studies. In addition to controlling protein turnover, ubiquitination has been known to regulate protein localization and enzyme activity. The effects of Ub E3 ligases on Rho GTPases translocalization and activities have not been revealed. Moreover, the ubiquitination may also influence the Rho GTPases interaction with their downstream molecules, such as Rho kinase and PAK1, in turn affecting Rho GTPases-mediated epithelial and endothelial barrier integrity.

## Future Directions

The regulation of cell junction protein turnover appears to be crucial for control of intercellular adhesion, junction dynamics, and barrier function. In this review, we described Ub E3 ligases and DUBs that mediate junctional protein endocytosis, ubiquitination, and recycling. Ub-dependent trafficking and turnover of these proteins rapidly regulate paracellular permeability and adapt to physiological variations. Despite accumulating evidences showing that targeting Ub E3 ligases and DUBs control epithelial and endothelial barrier integrity, the Ub E3 ligases and DUBs responsible for turnover of most key proteins in cell junctions have not been identified, especially for TJ proteins. Moreover, the molecular regulation of these enzymes needs to be investigated for better understanding their roles in cell junctions. Recent studies of ubiquitination and deubiquitination in regulation of Rho GTPases stability and turnover have unveiled additional important regulatory mechanisms. However, these studies focus mainly on RhoA and Rac1. Only a few studies center on Cdc42 stability even though Cdc42 plays an important role in regulating AJ assembly and endothelial barrier permeability (Kouklis et al., 2004; Dejana et al., 2008; Fukata and Kaibuchi, 2001; Broman et al., 2006). Lastly, DUBs in regulation of small GTPase remain largely unknown. Understanding these junctional proteins and their key regulators could lead to the development of inhibitors that target ubiquitination and proteasomal degradation which in turn could influence signal transduction, transcription, migration, proliferation, trafficking, and cytoskeleton dynamics in pathologicalc conditions. The majority of studies on regulation of cell-cell junction proteins turnover are in the cancer field, leaving the biological effects of these Ub E3 and DUBs on lung alveolar-capillary or blood-brain barrier relatively unknown. In summary, this review provides novel insight into how intercellular junction proteins and their regulator Rho GTPases interact with the UPS and DUBs system in maintenance normal barrier function and which targets might provide targets for therapeutic drugs.
